# Effect of hydrogen treatment temperature on the properties of InGaN/GaN multiple quantum wells

**DOI:** 10.1186/s11671-017-2109-6

**Published:** 2017-05-02

**Authors:** Yadan Zhu, Taiping Lu, Xiaorun Zhou, Guangzhou Zhao, Hailiang Dong, Zhigang Jia, Xuguang Liu, Bingshe Xu

**Affiliations:** 1 Key Laboratory of Interface Science and Engineering in Advanced Materials, Ministry of Education, Taiyuan University of Technology, 030024 Taiyuan, China; 2 Research Center of Advanced Materials Science and Technology, Taiyuan University of Technology, 030024 Taiyuan, China; 3 College of Chemistry and Chemical Engineering, Taiyuan University of Technology, 030024 Taiyuan, China

**Keywords:** InGaN, Hydrogen, Treatment temperature, Multiple quantum wells

## Abstract

InGaN/GaN multiple quantum wells (MQWs) were grown with hydrogen treatment at well/barrier upper interface under different temperatures. Hydrogen treatment temperature greatly affects the characteristics of MQWs. Hydrogen treatment conducted at 850 °C improves surface and interface qualities of MQWs, as well as significantly enhances room temperature photoluminescence (PL) intensity. In contrast, the sample with hydrogen treatment at 730 °C shows no improvement, as compared with the reference sample without hydrogen treatment. On the basis of temperature-dependent PL characteristics analysis, it is concluded that hydrogen treatment at 850 °C is more effective in reducing defect-related non-radiative recombination centers in MQWs region, yet has little impact on carrier localization. Hence, hydrogen treatment temperature is crucial to improving the quality of InGaN/GaN MQWs.

## Background

InGaN/GaN-based high-brightness light-emitting diodes (LEDs) have attracted tremendous attention over the past decade because of their applications in signage, back lighting, automotive headlights, and general illumination [1–5]. Despite of the great progress in materials and devices, there are still many unsolved issues related to the growth of InGaN/GaN multiple quantum wells (MQWs). One of the most serious problems is that the large lattice mismatch and low miscibility between InN and GaN result in the generation of several kinds of defects, such as alloy disordering, misfit dislocations, and stacking faults, which deteriorates the quality of MQWs [6, 7]. Generally, the typical growth temperature of InGaN quantum well (QW) for blue and green LEDs is below 800 °C to benefit indium incorporation [8, 9]. Meanwhile, the growth temperature of GaN quantum barrier (QB) is usually 50–150 °C higher than that of InGaN QW because of the low dissociation temperature of InN [6, 10]. Hence, the growth temperature of GaN barrier represents a compromise between the crystal quality of GaN and the In content in InGaN [11]. The lower growth temperature of GaN QB can result in poor crystal quality of MQWs [12, 13]. Moreover, in order to alleviate indium desorption or re-evaporation during the temperature ramp-up and GaN QB growth process, a thin low-temperature GaN cap layer (LT-cap) is usually deposited immediately after the growth of InGaN QW layer [14–17]. Nevertheless, the crystal quality of GaN LT-cap layer is very poor. It is reported that increasing the thickness of LT-cap layer can seriously degrade the optical quality of InGaN/GaN MQWs [18, 19]. Hence, the quality of InGaN/GaN MQWs is inferior in general. Given the key role of MQWs in LEDs, obtaining high quality MQWs will bring huge gains in device performance. Up to now, many methods have been proposed, such as growth of GaN barriers using hydrogen as carrier gas [20, 21], growth interruption [22, 23], indium treatment [24, 25], staggered QWs [26–28], InGaN barrier [29–31], and Al(In)GaN cap layer [32–34]. Recently, Ren et al. reported that full H_2_ treatment on GaN barrier layer can enhance the optical quality of InGaN/GaN MQWs [35].

In this paper, a small H_2_ flow was introduced to treat the QW/QB upper interface before barrier layer growth, and the effect of treatment temperature on the optical and structural properties of InGaN/GaN MQWs was investigated. It was found that H_2_ treatment at 850 °C is more effective in reducing defect density, as demonstrated by the remarkably improved room temperature photoluminescence (PL) intensity, and the decreased surface roughness and pit density.

## Methods

The InGaN/GaN blue emission MQW structures were grown on (0001) planar sapphire substrates by metal-organic chemical vapor deposition (MOCVD). The structure consisted of a 3.2-μm-thick nominally undoped GaN layer, and six pairs of InGaN/GaN MQWs with nominal 2.3-nm-thick InGaN wells separated by 10.5-nm-thick, lightly Si-doped (n-doping = 3 × 10^17^cm^−3^) GaN barriers. InGaN wells and GaN barriers were grown at 730 and 850 °C, respectively. A 1.0-nm-thick GaN LT-cap layer was deposited immediately after the growth of QW layer. Intervals without growth existed between each LT-cap and QB growth. Three samples with QW/QB upper interface treated at different conditions were prepared. Sample A, as the reference sample, employed pure N_2_ carrier gas during the temperature ramp-up process, while samples B and C were grown with 20 s H_2_ treatment, after each LT-cap layer growth but before the growth of each GaN barrier layer, at nominal treatment temperatures 730 and 850 °C, respectively. During the H_2_ treatment process, 200 sccm H_2_ was injected into the chamber.

High-resolution X-ray diffraction (HRXRD) measurement was performed using PANalytical Empyrean instrument to obtain the structure parameters. The PL properties were characterized by 325 nm He-Cd continuous wave laser, and the excitation power density was about 4.0 W/cm^2^. The temperature dependence of the luminescence spectra was measured from 10 to 300 K in a closed loop He cryostat. The luminescence was dispersed by a triple grating 50 cm monochromator and was detected by a GaAs photomultiplier tube using conventional lock-in technique. The surface morphology was studied by atomic force microscopy (AFM) (SPA-300HV) using tapping mode.

## Results and discussion

**Fig. 1:**
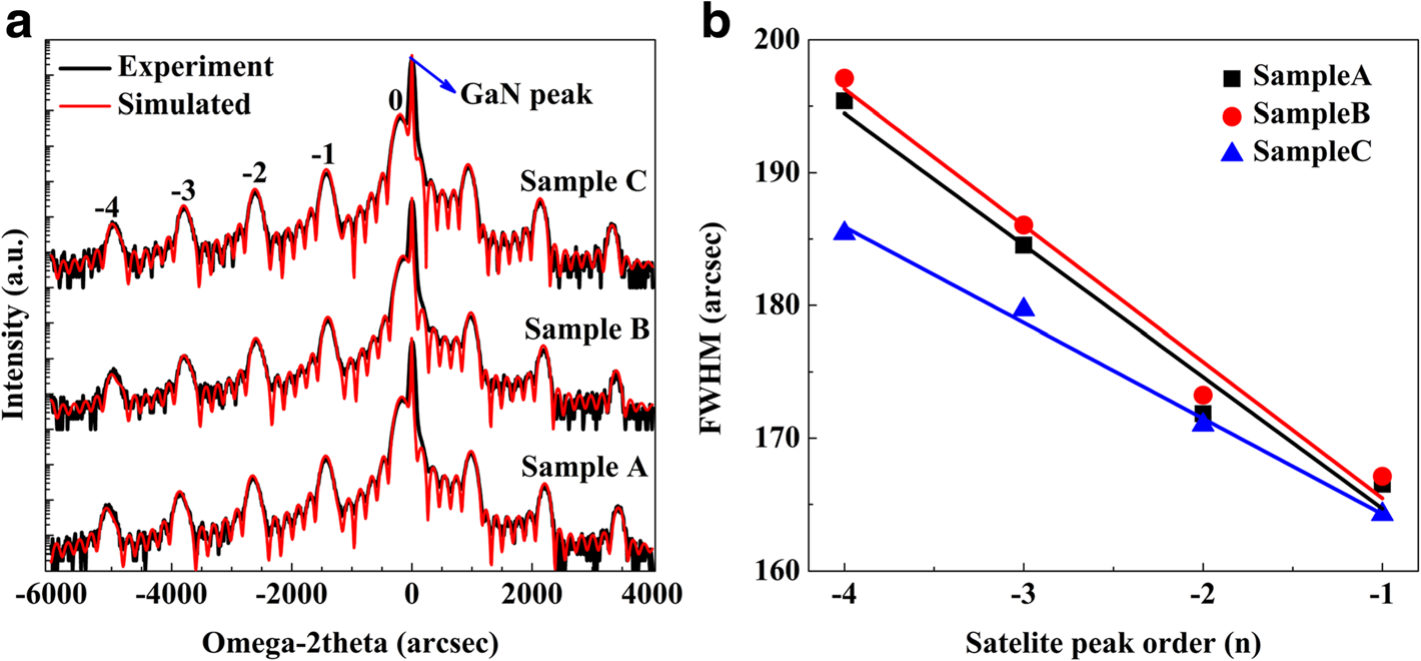
**a** HRXRD ω/2θ scanning curves and simulations of the samples. **b** The FWHM value as a function of satellite peak order and its linear fitting for the samples

Figure 1a shows HRXRD (0002) diffraction curves obtained by ω/2θ scans along the growth direction for the three samples. It can be seen that the simulation curves fit the experimental results very well. The satellite peaks up to the “−4th” order and Pendellösung fringes are clearly observed for the samples, indicating the fine periodic structure of MQWs, and the sharp interface between InGaN well and GaN barrier [36]. The average indium content and well thickness of QW layer obtained by fitting the measured curves for samples A, B, and C are 10.95% and 2.30 nm, 10.76% and 2.32 nm, and 11.11% and 2.36 nm, respectively. The period thickness of MQWs for the three samples is kept around 13.80 nm. The full widths at half maximum (FWHMs) of InGaN “−1st” diffraction peak for the three samples are very close, as shown in Fig. 1b. The almost unchanged structural parameters indicate that the 1.0-nm-thick cap layer can effectively protect InGaN QW layer during the following H_2_ treatment process. The interface roughness can be calculated by fitting the FWHMs of the XRD satellite peaks using the following equation [18, 37]:

1 $$ {\omega}_n={\omega}_0+{\left(1\mathrm{n}2\right)}^{1/2}\cdot \Delta {\theta}_m\cdot \frac{\delta}{\Lambda_0}\cdot n $$

where ω_n_ is the FWHM of the nth satellite peak, ω_0_ represents an intrinsic width of satellite peaks, Δθ_m_ is the angle spacing between the adjacent satellite peaks, and Λ_0_ is the period thickness of the InGaN/GaN MQWs. The slope of the fitting line is related to the interface roughness δ. The FWHM value as a function of satellite peak order is illustrated in Fig. 1b. It is obvious that sample C has a smaller absolute value of the slope, as compared with other two samples. Hydrogen treatment at 730 °C has little impact on interface roughness, as demonstrated by the little difference of slope value between samples A and B. These results indicate that interface modification is closely correlated with treatment temperature, and hydrogen treatment at higher temperature is beneficial to improving the interface quality.

**Fig. 2:**
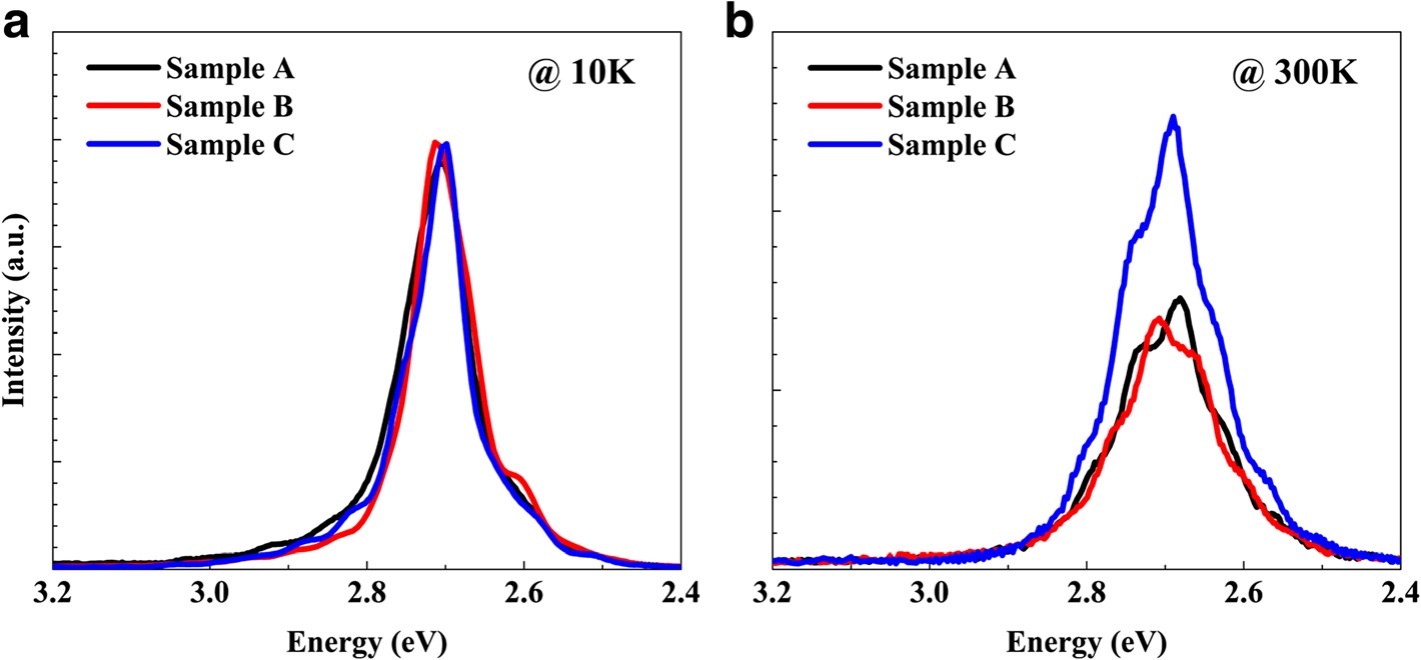
The measured PL spectra **a** at 10 K, and **b** at 300 K of all the samples

The measured low temperature (10 K) and room temperature (300 K) PL spectra of the three samples are shown in Fig. 2. The observed multiple peaks come from Fabry-Perot interference fringes at substrate/GaN/air interface. The PL intensity at 10 K of the three samples changes a little. The PL peak energy (FWHM) at 10 K obtained by Gaussian fitting for samples A, B, and C is 2.711 eV (110 meV), 2.704 eV (100 meV), and 2.708 eV (99 meV), respectively. The nearly unchanged PL peak energy further reveals that 1.0-nm-thick GaN cap layer can effectively protect InGaN QW layer from indium loss during the H_2_ treatment process. The PL peak energy (FWHM) at 300 K obtained by Gaussian fitting for samples A, B, and C is 2.697 eV (158 meV), 2.696 eV (156 meV), and 2.698 eV (149 meV), respectively. It can be seen that the PL intensity at 300 K for sample A is close to that of sample B, while sample C shows a significant enhancement. The remarkable increase in room temperature PL intensity and decrease in line width for sample C indicate that H_2_ treatment at 850 °C is more effective in improving the optical qualities.

**Fig. 3:**
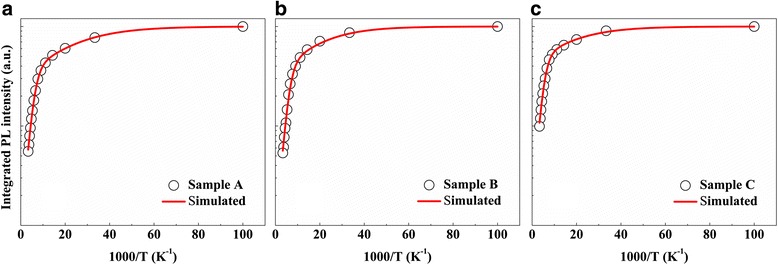
Temperature-dependent integrated PL intensity of the **a** sample A, **b** sample B, and **c** sample C

Figure 3 shows temperature-dependent integrated PL intensity of the three samples. It can be seen that the normalized integrated PL intensity shows monotonic decreasing behavior as the temperature increases, which can be well fitted using the following Arrhenius formula [38, 39]:

2 $$ I(T)={\left[1+{C}_1 \exp \left(-{E}_{A1}/{k}_B T\right)+{C}_2 \exp \left(-{E}_{A2}/{k}_B T\right)\right]}^{\hbox{-} 1} $$

where *I*(*T*) represents the normalized integrated PL intensity. The parameters *C*_1_ and *C*_2_ are two constants related with the density of non-radiative recombination centers in the samples. *E*_A1_ and *E*_A2_ are the activation energies corresponding to the non-radiative recombination process. *k*_B_ is Boltzmann’s constant. All the fitting results are listed in Table 1. It can be seen that the three samples have similar values of *E*_A1_ and *E*_A2_, meaning the unchanged types of non-radiative recombination centers after the interface treatment [40]. The values of *C*_1_ and *C*_2_ for sample B are close to those of reference sample A, indicating that the number of non-radiative recombination centers is nearly unchanged when H_2_ treatment is conducted at QW growth temperature. In contrast, as the hydrogen treatment is carried out at QB growth temperature, the values of *C*_1_ and *C*_2_ significantly decrease from 2.35 to 1.40, and 112.77 to 62.77, respectively. The smaller *C*_1_ and *C*_2_ for sample C reveal that H_2_ treatment at high temperature can significantly reduce the density of non-radiative combination centers in MQWs region. As aforementioned, the crystal quality of GaN LT-cap layer is inferior as a result of the low growth temperature, which means the density of non-radiative recombination centers is high in this layer [18, 19]. Impurities, threading dislocations, V-pits and other defects are widely regarded as non-radiative recombination centers in InGaN/GaN MQWs, which are detrimental to the radiative recombination of carriers [41–43]. Piner et al. reported that adding a small amount of hydrogen into the carrier gas can significantly reduce carbon and oxygen concentrations in InGaN alloy, because hydrogen atoms can interact either with hydrocarbon radicals or with hydroxyl radicals to form highly stable products (CH_4_, H_2_O) [44]. Hydrogen molecules thermally dissociate to hydrogen atoms at high temperature and the dissociation rate increases exponentially as the temperature rises [45]. Besides, the high temperature promotes the volatile products desorption and outdiffusion from GaN surface. Therefore, more carbon and oxygen impurities at LT-GaN surface can be removed at high temperature. Furthermore, impurities and indium atoms can easily segregate to and diffuse along the dislocations, V-pits, and stacking faults, because of the presence of dangling bonds or local stresses or electrical fields in these areas [46, 47]. It is reported that threading dislocations propagating through GaN buffer layer lead to the formation of V-pits in MQWs region, while using higher growth temperature or hydrogen as the carrier gas can reduce the density and size of V-pits [23, 48]. Recently, Yeh at al. reported that hydrogen treatment at high temperature can etch away threading dislocations (TDs) in GaN templates [49]. However, it is difficult to etch away TDs under low temperature (730 ~ 850 °C) and small hydrogen flow (200 sccm). The etching effect on the LT-GaN surface is very weak, as evidenced by the almost unchanged structural parameters of the MQWs. Nevertheless, hydrogen can react with indium atoms segregated at TDs to form volatile indium-hydride species, which desorbed rapidly at high temperature [50]. Hydrogen can also diffuse along the dislocation line and react with impurities during the treatment duration. Because the treatment temperature for sample C is 120 °C higher than that of sample B, more hydrogen atoms are available from H_2_ pyrolysis. Moreover, the reaction rates between hydrogen atoms and defects (impurities and indium atoms segregated at TDs) would increase fastly as the temperature rises. As a result, the abovementioned defects can be reduced more efficiently after the hydrogen treatment conducted at higher temperature, which consequently contributes to the remarkable improvement in optical qualities for sample C.

**Table 1:** Obtained fitting parameters: activation energies (*E*_A1_ and *E*_A2_), constants (*C*_1_ and *C*_2_), and localization energy (σ)

Sample ID	*C*_1_	*E*_A1_ (meV)	*C*_2_	*E*_A2_ (meV)	σ (meV)
Sample A	2.35	5.44	112.77	53.18	12.57
Sample B	2.16	6.46	124.84	54.72	12.34
Sample C	1.40	6.03	62.77	56.01	13.30

**Fig. 4:**
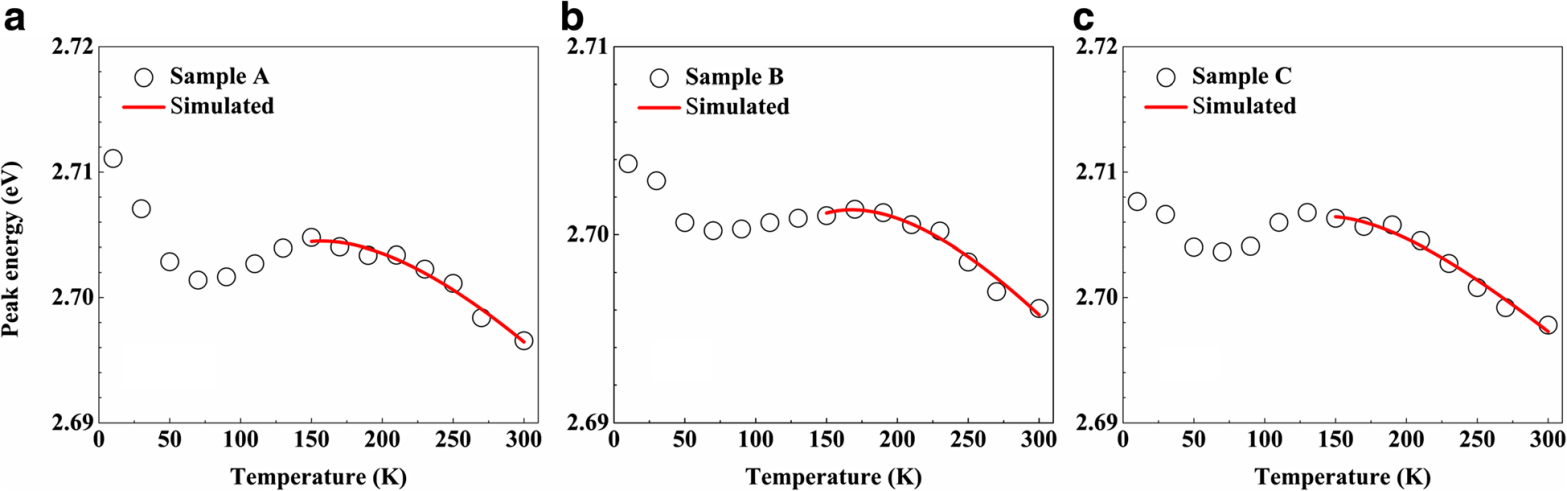
Temperature-dependent PL peak energy of the **a** sample A, **b** sample B, and **c** sample C

In order to elucidate the physical origin of the improvement in optical qualities, temperature-dependent peak energy of the three samples is shown in Fig. 4. All the samples exhibit S-shaped curves, which is a typical feature of the localization effect in InGaN/GaN MQWs structures [51]. The redistribution of carriers in the deep and shallow localized centers contributes to the first red-shift and then blue-shift of peak energy, as the temperature increases from 10 to 150 K. With further increase in the temperature to 300 K, the temperature induced band-gap shrinkage contributes to the red-shift of peak energy [52]. The localization energy (σ) can be obtained by fitting the temperature-dependent peak energy curve using Band-tail model [53, 54]. The obtained localization energy for samples A, B, and C is 12.57, 12.34, and 13.30 meV, respectively, as shown in Table 1. The very small change in the localization energy indicates the negligible impact of hydrogen treatment on the depth of carrier localization. This result is quite different from the work by Ren et al., in which the degree of carrier localization would be enhanced after hydrogen treatment [35]. In their work, hydrogen treatment was presented at the barrier surface by 1000 sccm H_2_, while other gases and precursors were switched off during the treatment. The difference may arise from that the hydrogen treatment here is conducted after the deposition of LT-cap layer and only 200 sccm H_2_ is added to the carrier gas to treat QW/QB upper interface. Under these conditions, the surface fluctuation caused by hydrogen treatment would be negligible. In other words, the undulating surface, favorable for the formation of phase segregation and quantum dot, is not satisfied [55, 56]. Consequently, the localization energy is almost unchanged after hydrogen treatment. Most importantly, these results verify that the enhancement of optical quality originates from the decreased density of defects in active region, rather than carrier localization.

**Fig. 5:**
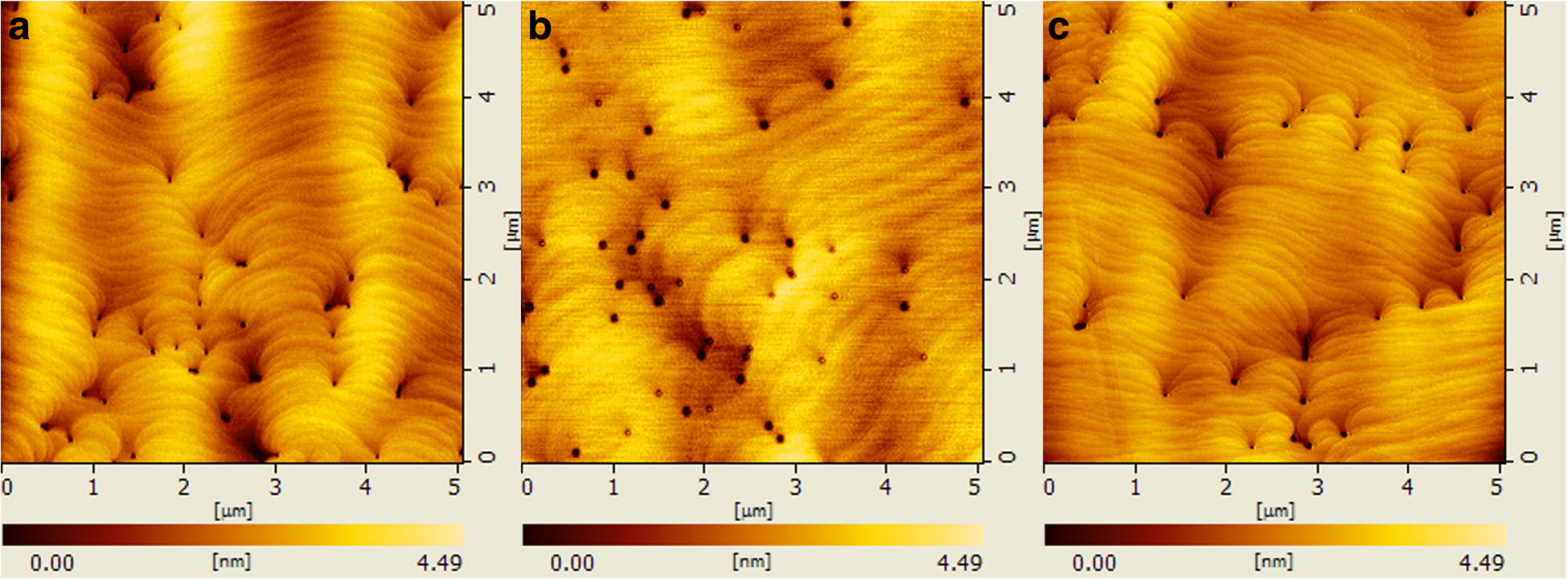
The AFM images (5 × 5 μm) of the **a** sample A, **b** sample B, and **c** sample C

The surface morphology of the samples tested by AFM is shown in Fig. 5. Atomic steps and pits are clearly observed in all samples. The calculated root mean square (RMS) surface roughness and pit density of the three samples are 0.619 nm and 1.96 × 10^8^ cm^−2^, 0.601 nm and 2.00 × 10^8^ cm^−2^, and 0.496 nm and 1.48 × 10^8^ cm^−2^, respectively. It is obvious that introducing hydrogen treatment at 850 °C is beneficial in reducing surface roughness and pit density, while hydrogen treatment at 730 °C has little impact on surface quality. The difference in surface qualities between samples B and C may originate from the more effective removal of impurities, indium atoms segregated at TDs, and pits in active regions by H_2_ under higher temperature, as aforementioned. As a result, the decreased defect density can enhance the two-dimensional growth of barrier layer and beneficial in forming flat surface and sharp interface between well and barrier layer [57, 58]. Consequently, both the surface and interface qualities are improved by employing hydrogen treatment at 850 °C.

## Conclusions

In summary, the effects of hydrogen treatment at different temperatures on optical and structural characteristics of InGaN/GaN MQWs are investigated. It is found that the degree of carrier localization is independent on hydrogen treatment, while hydrogen treatment at 850 °C can effectively reduce the non-radiative recombination centers in MQWs region. However, the sample with hydrogen treatment at 730 °C shows similar properties with the reference one. Hence, treatment temperature is an important parameter to improve the crystal quality of MQWs during hydrogen treatment process. Moreover, the physical origin of huge enhancement of room temperature PL intensity for the sample treated at 850 °C is the decreased defect density in MQWs accompanied by improved interface and surface qualities, rather than carrier localization.

## Abbreviations

AFM: Atomic force microscopy
FWHM: Full width at half maximum
HRXRD: High resolution X-ray diffraction
LED: Light-emitting diode
MOCVD: Metal-organic chemical vapor deposition
MQW: Multiple quantum well
PL: Photoluminescence
